# Identifying Gut Microbiome Features that Predict Responsiveness Toward a Prebiotic Capable of Increasing Calcium Absorption: A Pilot Study

**DOI:** 10.1007/s00223-024-01201-8

**Published:** 2024-04-24

**Authors:** Owen Ma, Arindam Dutta, Daniel W. Bliss, Cindy H. Nakatsu, Connie M. Weaver, Corrie M. Whisner

**Affiliations:** 1 Electrical Engineering, Arizona State University, 650 E Tyler Mall, Tempe, AZ 85281 USA; 2 Agronomy, Purdue University, 915 Mitch Daniels Boulevard, West Lafayette, IN 10587 USA; 3 Exercise and Nutritional Sciences, San Diego State University, 5500 Campanile Drive, San Diego, CA 92182 USA; 4 Health Solutions, Arizona State University, 500 N 3rd Street, Phoenix, AZ 85004 USA

**Keywords:** Bone health, Mineral metabolism, Adolescence, Prebiotic microbiome, Machine learning, Support vector machine, Calcium absorption

## Abstract

Previously, we demonstrated that prebiotics may provide a complementary strategy for increasing calcium (Ca) absorption in adolescents which may improve long-term bone health. However, not all children responded to prebiotic intervention. We determine if certain baseline characteristics of gut microbiome composition predict prebiotic responsiveness. In this secondary analysis, we compared differences in relative microbiota taxa abundance between responders (greater than or equal to 3% increase in Ca absorption) and non-responders (less than 3% increase). Dual stable isotope methodologies were used to assess fractional Ca absorption at the end of crossover treatments with placebo, 10, and 20 g/day of soluble corn fiber (SCF). Microbial DNA was obtained from stool samples collected before and after each intervention. Sequencing of the 16S rRNA gene was used to taxonomically characterize the gut microbiome. Machine learning techniques were used to build a predictive model for identifying responders based on baseline relative taxa abundances. Model output was used to infer which features contributed most to prediction accuracy. We identified 19 microbial features out of the 221 observed that predicted responsiveness with 96.0% average accuracy. The results suggest a simplified prescreening can be performed to determine if a subject’s bone health may benefit from a prebiotic. Additionally, the findings provide insight and prompt further investigation into the metabolic and genetic underpinnings affecting calcium absorption during pubertal bone development.

**Supplementary Information:**

The online version of this article contains supplementary material available 10.1007/s00223-024-01201-8.

## Introduction

The intestinal tract is inhabited by one of the most complex microbial communities and is believed to contribute considerable physiological capacity for human health [1]. The activities of gut microbes range from immune, metabolic, and nutrient uptake functions that are carried out by a gene pool that dwarfs that of the human genome by more than 450 times [2]. Although this community of bacteria, viruses, fungi, archaea, and protozoa remain relatively stable during adulthood, early life events in addition to various factors including geographical location, genetics, medication, physical activity, and diet use can alter the composition of the gut microbiome [3].

Both animal and human studies have highlighted the importance of gut microbes in preventing and treating osteoporosis [4–6]. Recently, we showed that prebiotic consumption positively influenced calcium absorption during early adolescence [7–9], a period when approximately $25\%$ of the adult skeleton is accrued [10]. This beneficial effect is thought to be mediated by the gut microbiome as changes in specific microbes correlated with enhanced calcium absorption [7, 8]. As osteoporosis risk is dependent on early-life health behaviors, an imbalance in calcium deposition and resorption in bone may increase the risk of osteoporosis later in life. Despite a body of literature that highlights the effectiveness of prebiotics on calcium absorption and bone density, two studies have suggested that not all individuals receive a benefit following prebiotic consumption [11, 12]. One of these studies [12] was completed in Malaysia with participant demographics similar to a previous Malaysian cohort characterized with lower diversity and abundance of beneficial microbes [13]. This lack of microbiome diversity leading to an absence of specific taxa may explain the null findings and highlights the importance of studying the gut microbiome in prebiotic and bone health studies.

The concept of responders and non-responders appears throughout the health literature and has been identified as an important area of study in the field of personalized medicine [14]. The gut microbiome is thought to mediate the benefits of prebiotics on bone mineral accretion, so evaluating differences in the gut microbiome by those who respond and do not respond to prebiotic consumption is important. The goal of this secondary data analysis was to evaluate differences in the baseline gut microbiome composition between Caucasian girls (12–14 y) who responded to prebiotics (experienced a $\ge$ 3% increase in calcium absorption [11] relative to control) and those who did not and whether baseline composition predicted responder status.

In this secondary data analysis, we employed machine learning to help us identify which gut microbial taxa were most informative of prebiotic responsiveness. Due to the costly yet sensitive and accurate assessment of calcium absorption provided by stable isotope methodologies, small sample sizes are sufficient to see treatment effects. However, combining these findings with gut microbiome data, which is heterogeneous with high sparsity across microbial taxa, infrequently provides the number of samples typically needed to leverage machine learning techniques. Furthermore, the dimensionality of the data is large relative to the sample population. These attributes introduce concerns of over-fitting and poorly representing the high-dimensional space, which together yield inaccurate, sensitive classifiers. Some initial dimensional reduction is imperative in this situation. A traditional technique may not be suitable in this scenario though. In particular, principal components analysis would not fare well because the data covariance matrix would be poorly estimated and have an unfavorable condition number due to insufficient samples. We carefully constructed our analysis to address these issues, including an alternative method for removing measurement redundancies.

## Materials and Methods

### Study Design and Data Collection

This study is based on data collected and published by Whisner et al. [7]. To briefly review the study design and outcomes of [7], participants each participated in three dietary interventions, composed of 0, 10, and 20 g/day of soluble corn fiber (SCF), administered in random order. During each intervention, participants consumed two half-doses of SCF daily in muffins and drink mixes for four weeks. Diets were otherwise unregulated. Intervention periods were separated by a four-week washout phase. Baseline and post-intervention microbiome compositions were measured by sequencing microbial DNA obtained from fecal samples via the V3–V4 region of the 16S rRNA gene. The Greengenes database was used as a basis for identifying microbial taxa present within samples [15]. Techniques for sequencing are described in further detail in [7]. Each sample was represented as a distribution of proportions, measuring the relative abundance of 221 taxa. We denote the column vector,

1 $$\begin{aligned} \textbf{x}_n = \begin{bmatrix} x_{n,1}&\cdots&x_{n,221} \end{bmatrix}^\text {T} \,, \end{aligned}$$

where element $x_{n,m}$ is the abundance of the *m*th taxon for the *n*th sample and $\Vert \textbf{x}_n\Vert _1=1$. Six samples (2 samples per intervention $\times$ 3 interventions) were collected from each participant. Fractional calcium absorption was assessed at the end of each intervention using a dual-stable isotope method [7]. Participants were deemed responsive to SCF if fractional calcium absorption increased by at least $3\%$ relative to control based on previous work [11]. We documented the mean fraction of calcium absorbed for each SCF dosage in Table 1.

**Table 1:** Average Fraction of Calcium Absorbed

Dosage (g/day)	Responder	Non-responder
0	0.3503	0.3840
10	0.4051	0.3484
20	0.4062	0.3471

For this analysis, only pre-intervention and post-placebo data were used to infer the effect of baseline gut microbiome composition on SCF responsiveness. Consequently, four of the six available samples were analyzed per subject to achieve the highest possible sample size for analysis. As documented in the parent paper [7], 28 participants completed all interventions. However, calcium absorption data were incomplete for three subjects, and microbial data were incomplete for another. Data from the remaining 24 subjects were analyzed in this study for a grand total of $N=96$ samples. Six of these 24 subjects responded to only one of the non-placebo doses. For this study, we relabeled the data, such that a participant was considered a responder if they responded to the 10 g, the 20 g, or both doses. Five subjects had only one outcome designation due to partially missing calcium absorption data. For each of those five subjects, overall responder status was determined based on the only information available. Under this labeling scheme, 19 of the 24 subjects were labeled responders, committing 76 of the 96 samples to the responder class. Each data vector $\textbf{x}_n$ had an associated label $d_n\in \{1,\, -1\}$, where 1 indicated responder and $-1$ represented non-responder.

### Pre-Processing

Our goal was to use machine learning to identify a concise set of gut microbial communities, where relative abundances of the constituents accurately predicted responder status. First, we pre-processed the data to simplify its handling and interpretation. Given some prior dimensional reduction, we produced a revised set of column vectors,

2 $$\begin{aligned} \tilde{\textbf{x}}_n = \begin{bmatrix} \cdots&x_{n,m}&\cdots \end{bmatrix}^\text {T} ,\, m\in {\mathcal {S}}, \end{aligned}$$

where ${\mathcal {S}}\subseteq \{1,\,\dots ,\,221\}$ is a set of indexes referring to the $|{\mathcal {S}}|=M$ remaining taxa. Then, we renormalized the data by evaluating

3 $$\begin{aligned} \textbf{y}_n=\frac{\tilde{\textbf{x}}_n}{\Vert \tilde{\textbf{x}}_n\Vert _1} \,, \end{aligned}$$

so that all vectors once again represented proper distributions. Afterward, we compressed the dynamic range of the data to accentuate subtle variations by computing

4 $$\begin{aligned} {\tilde{y}}_{n,m}=\sqrt{y_{n,m}} \,. \end{aligned}$$

Lastly, for each taxon $m\in {\mathcal {S}}$, we centered and normalized all ${\tilde{y}}_{n,m}$ according to the sample mean,

5 $$\begin{aligned} \mu _m=&\frac{1}{K}\sum _{n \in {\mathcal {A}}} {\tilde{y}}_{n,m} \,, \end{aligned}$$

and sample variance,

6 $$\begin{aligned} \sigma _m^2=&\frac{1}{K}\sum _{n \in {\mathcal {A}}} ({\tilde{y}}_{n,m}-\mu _m)^2 \,, \end{aligned}$$

where ${\mathcal {A}}$ is a set containing indexes of $|{\mathcal {A}}|=K$ designated training samples. We stored the standardized measurements in a new column vector,

7 $$\begin{aligned} \textbf{z}_n = \begin{bmatrix} \cdots&z_{n,m}&\cdots \end{bmatrix}^\text {T} ,\, m\in {\mathcal {S}}, \end{aligned}$$

where

8 $$\begin{aligned} z_{n,m}=\frac{{\tilde{y}}_{n,m}-\mu _m}{\sigma _m} \,. \end{aligned}$$

The feature vectors provided to the machine learning algorithm and resulting classifier were the set of $\textbf{z}_n$.

### Machine Learning

Our goal was to use a machine learning algorithm to develop a model that relates gut microbiome profiles to SCF responsiveness. We assessed the quality of that connection through the prediction accuracy. Support vector machines (SVMs) were used to construct predictive models and classify the pre-processed relative abundance data, $\textbf{z}$, into responder and non-responder classes. Presumably, each class could be represented in the feature space as separable clusters of data vectors. SVMs build hyperplanes that represent decision boundaries between groups. Specifically, we used a linear classification model. An important feature of a linear model, leveraged in subsequent analysis steps, was its ease of interpretation. The small sample size also made using more complex models prohibitive.

Given sets of training pairs $(\textbf{z}_n,\,d_n)$, where $n\in {\mathcal {A}}$, SVMs solved the following optimization problem:

9 $$\begin{aligned} \underset{\textbf{w},b,\varvec{\xi }}{\min }&\quad \frac{1}{2}\textbf{w}^\text {T}{} \textbf{w} +C \sum _{n \in {\mathcal {A}}} \xi _n \end{aligned}$$

10 $$\begin{aligned} \text {subject to}&\quad d_n(\textbf{w}^\text {T} \textbf{z}_n-b) \ge 1- \xi _n \end{aligned}$$

11 $$\begin{aligned}&\quad \xi _n\ge 0 \,. \end{aligned}$$

The boundary separating the two data classes was defined by $\textbf{w}^\text {T}{} \textbf{z}-b=0$, where $\textbf{w}$ was the hyperplane’s normal (column) vector and *b* was a bias term. Parameter $\xi _n$ in (9)–(11) provided slack to the *n*th training vector to allow convergence when the data were not perfectly separable. Cost parameter *C* in (9) controlled how much slack was provided. We restricted the amount of slack by setting $C=10$. After obtaining a model, $(\textbf{w},\,b)$, a test feature vector $\textbf{z}_n$, where $n \notin {\mathcal {A}}$, was classified by evaluating $\textbf{w}^\text {T} \textbf{z}_n-b$ and checking whether the resulting quantity fell above or below 0. We employed LIBSVM to construct and evaluate classifiers [16].

The dataset had a relatively small number of samples, and the representation from responders and non-responders was split 76 to 20. Small datasets complicate partitioning the dataset into training and testing sets without sensitivity to the outcome. Training on class-imbalanced datasets introduces decision biases, which would yield misleading performance and distorted interpretations of the features. To address these issues, we employed random partitioning to create balanced working datasets, five-fold cross-validation, and averaging performance measured over numerous retrials. Balanced working datasets were formed by first randomly selecting without replacement 20 samples from either class. We then created five partitions within each 20-sample pool and paired them to yield five balanced subsets. Each subset of eight samples took turns being the test set, where the remaining $K=32$ samples were used for training. Whenever ${\mathcal {A}}$ changed, we recomputed all $\mu _m$ and $\sigma ^2_m$ and subsequently re-evaluated all $\textbf{z}_n$. Redrawing datasets, repeating cross-validation, and averaging the performance alleviated sensitivity toward random sampling. We reshuffled the datasets $T=200$ times and repeated five-fold cross-validation each iteration to obtain an average performance. For further analysis, we also averaged the classifiers produced from every training phase by evaluating

12 $$\begin{aligned} \textbf{w}^\star =&\frac{1}{5T}\sum _{t=1}^{5T}{} \textbf{w}_t\end{aligned}$$

13 $$\begin{aligned} b^\star =&\frac{1}{5T}\sum _{t=1}^{5T} b_t \,, \end{aligned}$$

where *t* indexes the iteration. Reducing the amount of slack allowed for greater consistency after averaging.

### Dimensional Reduction

**Fig. 1:**
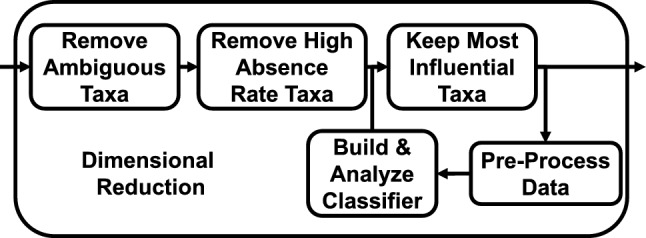
Overview of the dimensional reduction process. Measurements were first removed prior to learning based on the ambiguity of microbial classification and high absence rates. Additional measurements were removed by analyzing classifiers built from the remaining information and determining which measurements were most important

We hypothesized that a significant portion of the taxa did not strongly impact responsiveness. We employed three different techniques to remove redundant information within the gut microbiome abundance profiles. The dimensional reduction process is summarized in Fig. 1. We assessed the impact of removing information using the outcomes of cross-validation testing.

#### Taxon Ambiguity

The first reduction step disqualified from analysis taxa with the “Other” designation. Portions of the data that were unable to be definitively classified into a particular taxon were given this name. We hypothesized that these classes may not be specific enough to reveal distinguishing characteristics of the gut microbiome profile. Without any information removal, classifiers achieving 83.2% ± 6.3% accuracy were constructed. We deterministically removed 23 of these ambiguous taxa listed in Table S1 from the dataset, leaving information from 198 taxa to leverage. Prediction accuracy afterward was 84.1% ± 6.6%. The small change in accuracy indicated that no crucial pieces of information were lost.

#### Absence Rate

Several taxa listed accounted for 0% of every sample’s contents (i.e., for some taxon *m*, the abundance $x_{n,m}=0$ $\forall n$). There was no way to differentiate between groups based on the abundance of these particular taxa, so we removed them from analysis.

We relaxed this rule to disqualify additional taxa when they were almost always absent. If a taxon had a different absence rate based on responder status, then this feature may be advantageous to retain. Alternatively, if a taxon was absent with the same high rate across groups, then we assumed the relative abundance of that taxa was not informative. We defined the absence rate of taxon *m* as the fraction of samples within a responder group where $x_{n,m}=0$. If a taxon’s absence rate for both classes exceeded some threshold, then it was disqualified from the analysis.

#### Decision Influence

The last dimensional reduction technique disqualified taxa from the final analysis if they did not strongly influence the classifier’s decision-making. Determining each taxon’s influence involved producing an average classifier $(\textbf{w}^\star , b^\star )$ given just the two initial dimensional reduction steps, outlined in Sect. 2.4.1 and Sect. 2.4.2, and studying its construction. Interpreting how these SVM models interacted with the features was straightforward because we employed a linear classifier, where each coefficient in $\textbf{w}$ directly affected the abundances of a single taxon. A taxon’s influence was inferred by the amount of gain the classifier applied to its associated feature versus others. Facilitated by standardizing the measurements, we assumed that a classifier would emphasize certain information over others through amplification, suggesting that the abundances of those taxa were more correlated with responder class than others. A vector of gains was defined as

14 $$\begin{aligned} \textbf{g}= \begin{bmatrix} \cdots&|w_m^\star |&\cdots \end{bmatrix} ,\, m\in {\mathcal {S}}\,. \end{aligned}$$

We then assigned a rank for each taxon’s influence by sorting the gains according to the convention,

15 $$\begin{aligned} g_{o^{-1}(1)} \ge \dots \ge g_{o^{-1}(r)} \ge \dots \ge g_{o^{-1}(M)} \,, \end{aligned}$$

where $o^{-1}(r)$ maps the ranking *r* one-to-one back to a corresponding taxon ID $m\in {\mathcal {S}}$.

We disqualified communities from further analysis if their level of influence fell below some threshold and updated the set ${\mathcal {S}}$. This technique relied on properly standardizing each measurement, so some risk was involved when evaluating (8) because the distributions of these measurements are not well understood. We formed simplified data vectors from abundance measurements of the remaining taxa. This information was pre-processed and utilized in a second stage of multi-trial cross-validation to validate its utility.

#### Parameter Selection

**Fig. 2:**
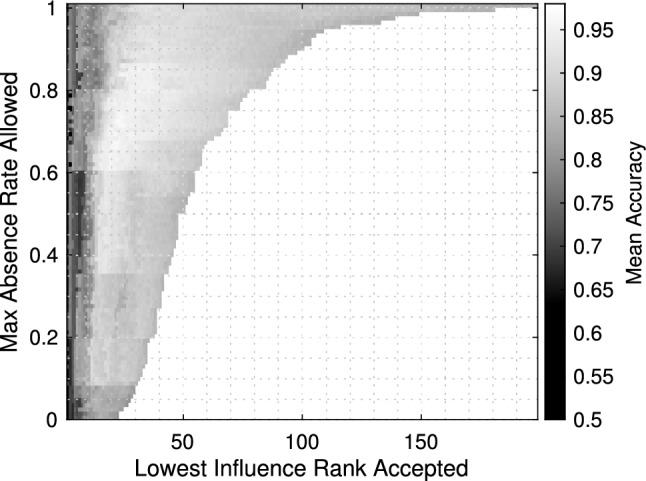
Average classification accuracy as a function of the parameter pairs used in dimensional reduction. A swath of parameter pairs yielded proficient prediction, indicating the processing chain was not sensitive to parameter selection. Several methods were employed to determine a suitable pair using this surface

Two of the three dimensional reduction techniques required threshold parameters to be set. These parameters included the maximum absence rate tolerated and the lowest rank of influence accepted. We performed a joint search, where the entire multi-trial cross-validation procedure was repeated while supposing values for each threshold.

Sweeping over all possible pairs yielded a surface of average classification accuracy shown in Fig. 2. When examining the surface, we observed regions of comparable performance, which indicated there were many viable choices. Our analysis was robust toward the specific selection of thresholds, which was especially important in this small sample size setting.

Several approaches were considered to select a parameter pair, such as choosing the mean accuracy maximizing thresholds. We pursued a technique that prioritized dimensional reduction. We chose the pair of parameters that yielded the smallest final dimensionality while maintaining some minimum level of classification accuracy. When requiring accuracy greater than or equal to 96%, we determined that taxa exceeding an absence rate of 60.42% should be removed (leaving 56 taxa) and only the 19 most influential taxa should be kept after assessing how each taxa contributed to classification.

Taxa disqualified due to high absence rates are listed within Table S2, and those removed due to weaker decision influence are listed in Table S3. Using the dimensional reduction procedure with the selected parameters allowed the SVM to produce classifiers that achieved 96.0% ± 3.1% accuracy. The dimensional reduction and subsequent prediction performance achieved indicates that a small subset of taxa were correlated with SCF responsiveness.

### Identifying Differences in Gut Microbiome Profiles

After identifying a concise subset of taxa that accurately predicted responder classes, we attempted to determine what characteristics in these simplified gut microbiome profiles predicted responsiveness. Presumably, differences in the joint distributions conditioned upon responder class would be identifiable provided the correlation established by the SVM model. Even though we reduced the dimensionality significantly, it was still large enough to complicate interpreting the relationships between the various taxa. We examined the differences in terms of marginal distributions of each taxon’s abundance conditioned upon the responder class. Given the small sample population, this method was perhaps one of the few viable options to identify taxa abundance trends that differed between classes. Descriptive statistics were not necessarily appropriate here because the distributions were often complex.

For each taxon, we compared the empirical distribution of its square root abundance, ${\tilde{Y}}_m$, given that the measurement came from a responder, $\text {Pr}({\tilde{Y}}_m|D=1)$, with the same given the measurement came from a non-responder instead, $\text {Pr}({\tilde{Y}}_m|D=-1)$. Random variable *D* represents the subject group. Caution must be taken when examining the taxa one by one because relationships between taxa that may be indicators of responsiveness are obscured.

## Results

### Influential Taxa

We identified communities whose relative abundance correlated with an eventual increase in calcium absorption. Classification was accurate when using relative abundances of the taxa listed in Table 2. Square brackets around names indicate a taxon that must be reclassified. These 19 communities most strongly influenced the classifier’s decision-making. Developing a ranking involved some stochasticity due to random partitioning. For other runs of the full analysis, lower-ranked taxa within Table 2 may swap places with taxa included in Table S3. Consequently, Table 2 should not be treated as a definitive list of taxa that solely predicted responsiveness.

**Table 2:** Most Influential Taxa

Rank	Previous	Name
1	1	Lachnospiraceae *Roseburia*
2	7	Lachnospiraceae *[Ruminococcus]*
3	5	Unclassified Clostridiaceae
4	8	Ruminococcaceae *Oscillospira*
5	6	Unclassified Coriobacteriaceae
6	17	Carnobacteriaceae *Granulicatella*
7	11	[Odoribacteraceae] Odoribacter
8	12	Veillonellaceae *Megamonas*
9	2	Lachnospiraceae *Coprococcus*
10	10	Lachnospiraceae *Anaerostipes*
11	4	Lachnospiraceae *Dorea*
12	3	Veillonellaceae *Megasphaera*
13	19	Coriobacteriaceae *Collinsella*
14	9	Unclassified Rikenellaceae
15	14	Christensenellaceae *Christensenella*
16	15	Veillonellaceae *Veillonella*
17	16	Unclassified Enterobacteriaceae
18	18	Veillonellaceae *Dialister*
19	13	Unclassified Peptostreptococcaceae

Analyzing the structure of the average classifier built using the fully reduced data revealed a reordered ranking of influence. Reordering tended to occur due to the additional taxon pruning, which changed the learning space. Both the reordered and previous rankings are listed with the remaining taxa in Table 2.

### Verifying the Dimensional Reduction Methodology

Additional variants on feature selection were implemented to help verify our approach and results. We performed the multi-trial cross-validation procedure using data vectors composed only of abundance measurements of taxa given the “Other” designation. We ranked their decision influence and determined the classification accuracy using various blocks of 19 adjacently ranked taxa. The maximum accuracy achieved was 55.9% ± 7.5%, indicating that the removed taxa were not strongly informative.

Similarly, we performed the complete multi-trial cross-validation process starting with only taxa that were removed based on absence rate. From the ranking, we also chose blocks of 19 taxa to assess their correlation with responsiveness. The classifiers in this particular experiment achieved at most 54.8% ± 3.4%, indicating a high likelihood that crucial information was not ignored.

**Fig. 3:**
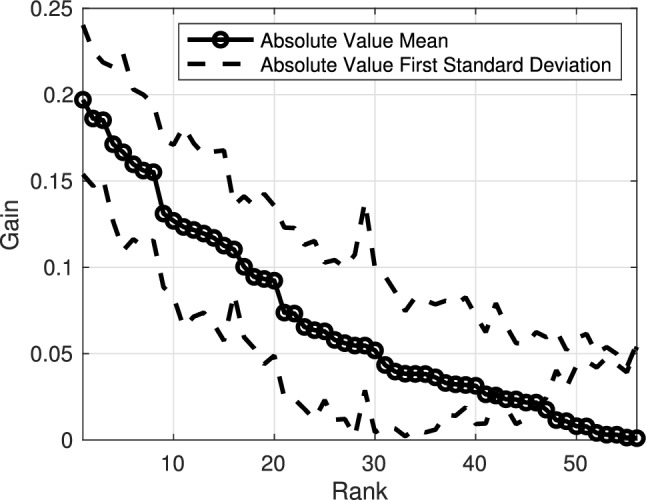
Classifier gain structure for measurements of each taxon remaining after the first two dimensional reduction steps (taxon ambiguity and absence rate). Differences in the average gains indicate that the classifier weighed information regarding certain taxa more heavily than others

We also confirmed that our method for interpreting classifier construction was sensible. Gains $\textbf{g}$ computed from $\textbf{w}^\star$ and arranged from largest to smallest are shown in Fig. 3. The average classifier clearly weighed certain pieces of information more significantly than others.

**Fig. 4:**
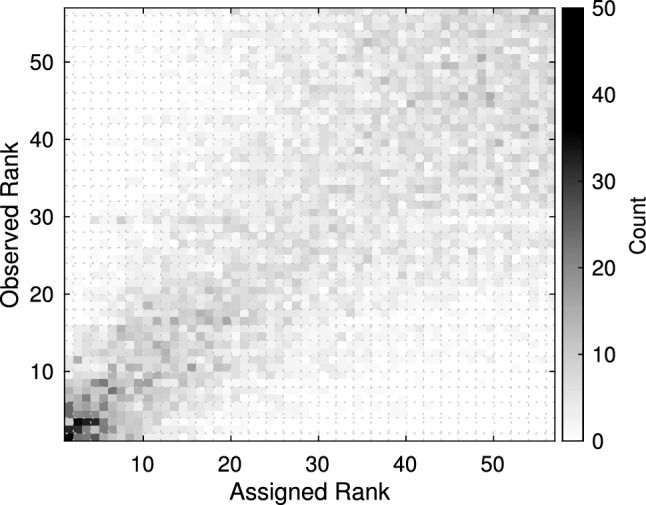
Histograms of the rankings each taxon could receive after every round of five-fold cross-validation illustrate how consistent the ordering methodology was over all retrials. Each column is a histogram for a particular taxon. A strong diagonal structure would indicate consistency. Taxa that were ultimately assigned a higher rank, were more likely to be placed into the same neighborhood despite redrawing training datasets

To assess the consistency and reliability of our methodology, we formed histograms of observed rankings for each taxon and present them all together in Fig. 4. Each column is associated with one taxon. Columns are arranged by their corresponding taxon’s assigned ranking. Ideally, the histogram should show a strong diagonal structure, which would indicate that the ordering across trials was consistent with the ranking assignments. We observed that rankings across cross-validation trials were more consistent in higher influence taxa than lower influence taxa.

**Fig. 5:**
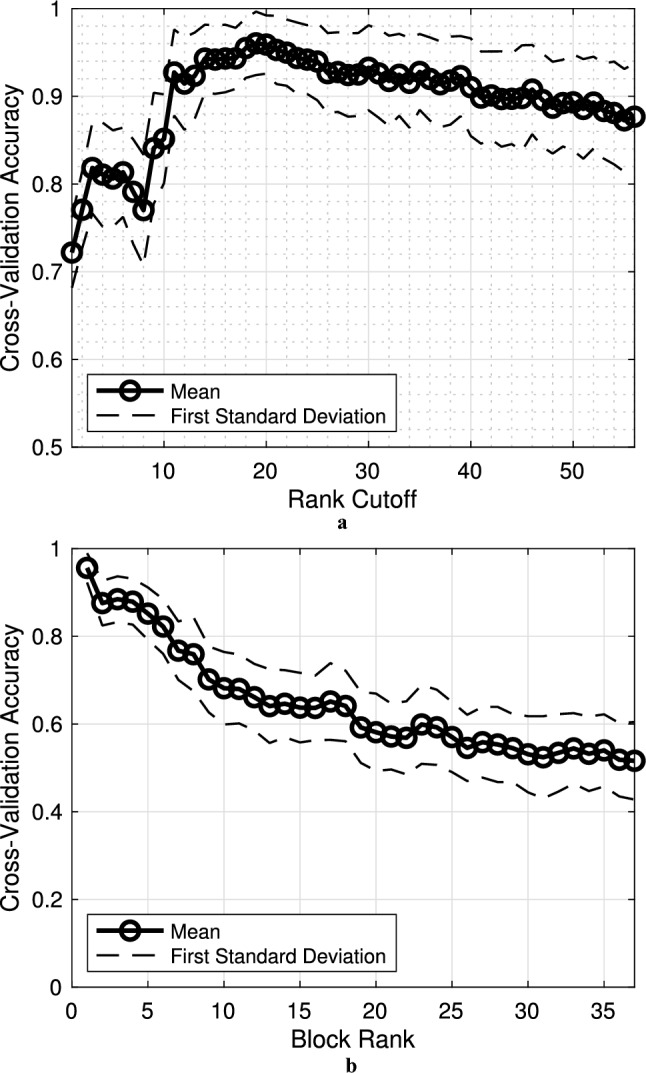
Determining the optimal reduction in measurements and verifying the sensibility of assigned taxa rankings. **a** Classifier accuracy was recorded when varying the number of rank-ordered taxa retained. Prediction was most accurate when retaining just the top 19 rank-ordered taxa. **b** Prediction accuracy was assessed as different subsets of 19 sequentially ranked taxa were used for training and testing. Performance generally degraded as more lower-ranked taxa were swapped in for higher-ranked taxa. By block 20, where no information about the top 19 ranked taxa remained, prediction accuracy was on average 58.1%

Findings demonstrated the benefit of trimming information based on decision-making influence. Figure 5a presents the cross-validation accuracy as a function of a cut-off on the least accepted influence, where taxa ranked below were removed before the second evaluation stage. The previous ranking column in Table 2 corresponds to the ordering used in Fig. 5a. The accuracy when the cut-off was at 56 (i.e., when taxa have only been removed if they were ambiguous or based on absence rate) was 87.7% ± 6.0%. We observed that as the threshold grew and more taxa were removed (moving from right to left), the average performance increased by another 8.3%. Increasing the threshold past the point of maximal performance, where classifiers achieved 96.0% ± 3.6% using abundance measurements from just 19 taxa, yielded diminishing returns. Classifier accuracy remained proficient even when the feature space decreased to the top 11 ranked taxa but fell significantly beyond that point.

We further corroborated the ordering strategy by examining classifier accuracy when using measurements from ranked blocks of taxa. Sweeping in 19-taxa blocks across the ordered list (i.e., taxa ranked $r=R,\,R+1,\,\dots ,\,R+18$, where *R* was the block number) yielded the curve in Fig. 5b illustrating the change in accuracy (left to right) as more influential taxa were gradually swapped for less influential ones. Performance generally degraded when using blocks of taxa that progressively decreased in influence, which indicated that the taxa were ranked appropriately. Using the 19 most influential taxa achieved the maximum classification accuracy (95.6% ± 3.3% in this particular run of trials). By the 20th block, none of the taxa listed in Table 2 were retained, and just 58.1% ± 8.8% accuracy was achieved. The characteristics of Fig. 5a and b indicated that the improvement in accuracy was due not only to dimensionality reduction but also to the specific information associated with the most influential 19 taxa.

One concern that arises during machine learning is that the accuracy achieved is a fluke. To help support that the accuracy achieved was not coincidental, we performed the second-stage multi-trial cross-validation process with randomly permuted training labels, which produced false classifiers. We tested the false classifiers with correctly labeled data and achieved 49.9% ± 8.4% accuracy, indicating that the models are in fact utilizing relevant information embedded within the data.

### Differences in Gut Microbiome Profiles

Empirical distributions of square root abundances, conditioned on responsiveness, $\text {Pr}({\tilde{Y}}_m|D=1)$ and $\text {Pr}({\tilde{Y}}_m|D=-1)$, for each taxon *m* within Table 2 are plotted as overlaid pairs in Fig. S1. The ordering of the plots corresponds with the updated ranking listed in Table 2. Consider the distributions of Lachnospiraceae *Roseburia* plotted in Fig. ﻿6 that exhibited the clearest distinction between classes in terms of relative abundance. The distributions overlapped, but their peaks and most observations were well separated by class. The median square root *Roseburia* abundance was 0.1820 for responders and 0.3648 for non-responders with more non-responders having a greater relative abundance. *Roseburia* influenced the classifier’s decisions more than all other taxa according to Table 2. Despite these observations, this figure provides an incomplete picture of how gut microbiome composition related with SCF responsiveness. Caution should be taken when interpreting individual distributions in Fig. S1 because the relative abundances of all 19 taxa should be examined jointly as taxa interpretation requires the context of others.

**Fig. 6:**
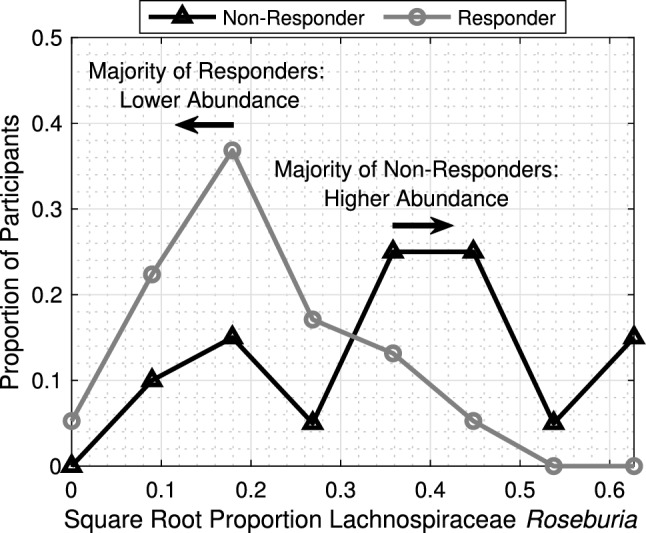
Empirical distributions of square root sample proportion of *Roseburuia* conditioned upon responder class. *Roseburuia* abundance had a particularly strong correlation as compared to other taxa. Non-responders tended to have a higher relative abundance of *Roseburuia* than responders

## Discussion

We identified a set of 19 gut microbial taxa, much smaller than the overall community ($M=221$) detected within samples, whose relative abundances were strongly correlated with responsiveness toward SCF. This analysis was based on baseline characterizations of gut microbiomes in a free-living context; therefore, our methodology was to some extent robust toward varying diets and environments. This result suggests that we only need to poll abundance for a select number of gut microbial taxa to determine if SCF will contribute to increased calcium absorption for a particular subject.

Previously, we performed simple pairwise comparisons to understand how microbial taxa abundance differed by SCF dose. Several of the microbial taxa identified aligned with the 19 most influential taxa resulting from SVM predictive modeling. These included members from the families Lachnospiraceae (*Anaerostipes* and *Dorea*) and Clostridiaceae and genera *Dialister*, *Megamonas*, and *Ruminococcus*. Interestingly, five of the 19 influential taxa (*Roseburia*, *Ruminococcus*, *Coprococcus*, *Anaerostipes*, and *Dorea*) identified with SVM were from Lachnospiraceae, a family known as prominent producers of the short-chain fatty acid butyrate in the human gut [17]. Previously, we observed no treatment differences in fecal butyrate concentrations but did note the significantly lower abundance of *Dorea*, *Ruminococcus*, and *Anaerostipes* following SCF consumption relative to control. Although not consistent across all Lachnospiraceae genera, SVM results suggested that a greater proportion of responders had higher baseline abundance of *Coprococcus*, *Ruminococcus*, and *Dorea*, but not *Roseburia* and *Anaerostipes*. This aligns with previous work suggesting that Lachnospiraceae members, such as *Roseburia*, *Dorea*, and *Ruminococcus*, are present at baseline but decrease upon SCF exposure or consumption [18, 19].

With advancements in science and medicine leading to precision health approaches for inflammatory conditions, such as cancer and cardiovascular disease, the promising effects of prebiotics on calcium absorption and bone health outcomes merit greater study. To date, few researchers have evaluated how calcium metabolism and skeletal benefits relate to the natural variation observed for prebiotic response, especially when considering the involvement of the gut microbiome. One study in postmenopausal women reported greater prebiotic responses in postmenopausal women with lower baseline bone mineral density (BMD) following consumption of oligofructose-enriched inulin [20]. Another study found that oligofructose-enriched inulin elicited differential responses, with only two-thirds of the study cohort responding ($\ge$3% increase in calcium absorption, similar to the present study) to the intervention [11]. Exploratory analyses revealed a prebiotic-gene interaction with responders being significantly more likely to harbor one or more dominant alleles (*FF* or *Ff*) for the *Fok1* gene [21]. Additionally, about 70% of the additional calcium absorption observed occurred in the colon, implicating lower gut mechanisms in understanding responder status [22]. None of the above-mentioned studies included gut microbiome profiling, making it difficult to know if the gut microbiome has similar influences on calcium absorption when different fibers are consumed.

The clinical and public health relevance of this work remains narrow as the time requirements and cost-prohibitiveness of clinical trial management combined with 16S rRNA gene sequencing have limited the number of clinical trials that are available for comparison. Nonetheless, it is important to compare our findings against other prebiotic fibers. Inulin, a heavily studied prebiotic for bone health was recently evaluated with the gut microbiome in a pilot study of 12 healthy adults [23]. This study reported increased abundance of *Streptococcus* and *Anaerostipes hadrus* in the inulin group compared to calcium supplement only, while *Bifidobacteria*, *Anaerostipes hadrus*, and *Roseburia intestinalis* were more abundant in the inulin group relative to a combined calcium and inulin group. Galactooligosaccharides (GOS) [9] have also been shown to increase the abundance of *Bifidobacteria* but these results were from quantitative PCR and did not account for the presence of other taxa which may also be important for calcium absorption. Interestingly, this study was also a dose-response with only the low dose (5 g/day vs. 10 g/day) of GOS eliciting a positive effect on *Bifidobacteria* abundance and calcium absorption. This highlights the importance of studying different prebiotics but also varying doses when evaluating the role of gut microbes on bone health.

Although this study demonstrated a strong correlation between specific microbial taxa and calcium absorption following SCF consumption, we acknowledge several limitations. The primary issue was the small number of participants in the parent study and subsequently, small number of samples available for analysis. Due to the heterogeneity of the gut microbiome across individuals, the 16S rRNA gene sequencing data had significant variation which could make reproducing these findings challenging with similar human datasets. Findings for SCF may not translate to other prebiotic fibers which have diverse structures and may interact with different microbial taxa to elicit a benefit to bone. The dataset also had a significant class imbalance where non-responders (the minority of the subjects) were not well represented within the measurement space. Simply having more data may have yielded a different set of results. Identifying distinguishing trends in the relative abundances was complicated by this issue too. Further, subjects that participated in the study were from a narrow demographic making extrapolation to non-White populations challenging. Consequently, a risk of over-fitting to this particular dataset inevitably arose. In the future, conducting a similar study with a more inclusive set of subject backgrounds may better reveal what gut microbiome distributions are strong correlates of responsiveness toward SCF across diverse populations.

A more controlled study may yield a different conclusion than the one resulting from this analysis, as studying free-living individuals may have increased variability and noise in the data. However, studying the data recorded on people as they naturally live and consume food adds real-world applicability and suggests the utility of this analysis in a variety of clinical and community health settings. Aside from iterating upon our study methodology, the results motivate continued research as well. Further investigation into the identified taxa in Table 2 and microbial community members that interact with them, especially those that are affected by SCF, should be conducted. One question that arises is the possibility of cultivating gut microbiome conditions that are more responsive to SCF given baseline makeups. Further along this direction, the results prompt examining how gut microbiome profiles (with particular respect to SCF non-responders) may indicate responsiveness toward other dietary fibers or supplements.

## Conclusion

Ensuring sufficient calcium absorption at a young age is beneficial to long-term bone health and well-being. A previous study by Whisner et al. determined that incorporating SCF into diets was effective in increasing calcium absorption in a majority of the study population. In this study, we investigated how baseline gut microbiome makeup in a free-living context may predict SCF responsiveness. We identified a concise subset of taxa whose relative abundances were correlated with responsiveness using machine learning. The majority of these taxa are known producers of short-chain fatty acids which are mechanistically important for enhancing calcium absorption. To accomplish this, we developed a methodology for controlling classifier construction such that we can interpret how it makes decisions and assess which microbial taxa most influence prediction. The results suggest that a simplified prescreening can be performed to determine if a subject may benefit from incorporating SCF into their diet. Additionally, the findings provide insight and prompt further investigation into the pathways that affect calcium absorption during critical periods of growth and osteoporosis prevention. As the desire for personalized nutrition increases among the public and 16S rRNA gene sequencing costs continue to decrease, the applicability of such predictive modeling will grow and guide the selection of prebiotics or fiber-rich foods that are most effective for individuals. It is also possible that as other prebiotic fibers undergo similar study and specific core microbial taxa are identified, affordable and more rapid testing techniques (e.g., quantitative PCR) can be implemented instead of 16S rRNA gene sequencing. This would greatly enhance the clinical utility of such analyses for both preventative care and disease treatment.

### Supplementary Information

Below is the link to the electronic supplementary material.

Electronic supplementary material 1 (PDF 165 kb)

## Data Availability

Not applicable.
