# Supplementary material for: Identifying Gut Microbiome Features that Predict Responsiveness Toward a Prebiotic Capable of Increasing Calcium Absorption: A Pilot Study
Source: Calcif Tissue Int. 2024 Apr 24;114(5):513–23. doi: 10.1007/s00223-024-01201-8 (PMC11061023; doi:10.1007/s00223-024-01201-8)
Supplement: Supplementary file 1 — Electronic supplementary material 1 (PDF 165 kb) [file 223_2024_1201_MOESM1_ESM.pdf]

Identifying Gut Microbiome Features that Predict Responsiveness toward a Prebiotic Capable of Increasing Calcium Absorption: A Pilot Study

Owen Ma[1*], Arindam Dutta[1], Daniel W. Bliss[1], Cindy H. Nakatsu[2], Connie M. Weaver[3], Corrie M. Whisner[4]

[1*]Electrical Engineering, Arizona State University, 650 E Tyler Mall, Tempe, 85281, AZ, USA.
[2]Agronomy, Purdue University, 915 Mitch Daniels Boulevard, West Lafayette, 10587, IN, USA.
[3]Exercise and Nutritional Sciences, San Diego State University, 5500 Campanile Drive, San Diego, 92182, CA, USA.
[4]Health Solutions, Arizona State University, 500 N 3rd Street, Phoenix, 85004, AZ, USA.

*Corresponding author(s). E-mail(s): owenma@asu.edu;
Contributing authors: adutta7@asu.edu; d.w.bliss@asu.edu; cnakatsu@purdue.edu; cmweaver@sdsu.edu; cwhisner@asu.edu;

Supplementary Materials

Tables

We document in Table S1, Table S2, and Table S3 communities whose relative abundances were removed from analysis. The first dimensional reduction step removed portions of the sample that were unable to be classified into a taxon within one of the more general communities listed in Table S1. Taxa removed based on their absence rate from either subject group are listed in Table S2. Lastly, taxa removed due to having a weaker influence on predictions are listed in Table S3. As discussed in Section 3.1, due to the stochastic nature of the analysis and decisions made regarding parameter selection, swapping taxa between Table 2 to Table S3 or moving some from one to the other is plausible.

Table S1 Ambiguous Taxa

Name	
Other Clostridiales	Other Rikenellaceae
Other [Mogibacteriaceae]	Other Rhizobiales
Other Unassigned	Other Ruminococcaceae
Other Oxalobacteraceae	Other Clostridiaceae
Other Enterobacteriaceae	Other Peptostreptococcaceae
Other Pasteurellaceae	Other Bacteroidales
Other Lachnospiraceae	Other Bacillales
Other [Tissierellaceae]	Other Firmicutes
Other Bifidobacteriaceae	Other Burkholderiales
Other Enterococcaceae	Other Coriobacteriaceae
Other Veillonellaceae	Other Intrasporangiaceae
Other Brucellaceae	

Table S2 Absence Rate Taxa

Name (Family *Genera*)	
Methanobacteriaceae *Methanobrevibacter*	Unclassified mb2424
Unclassified Actinomycetaceae	Actinomycetaceae *Mobiluncus*
Actinomycetaceae *Varibaculum*	Brevibacteriaceae *Brevibacterium*
Dermabacteraceae *Brachybacterium*	Gordoniaceae *Gordonia*
Microbacteriaceae *Cryocola*	Unclassified Micrococcaceae
Micrococcaceae *Kocuria*	Micrococcaceae *Micrococcus*
Micrococcaceae *Rothia*	Mycobacteriaceae *Mycobacterium*
Unclassified Bifidobacteriaceae	Bifidobacteriaceae *Scardovia*
Coriobacteriaceae *Atopobium*	Unclassified Bacteroidales
Porphyromonadaceae *Dysgonomonas*	Porphyromonadaceae *Porphyromonas*
Unclassified RF16	Unclassified S24-7
Unclassified [Paraprevotellaceae]	[Paraprevotellaceae] *CF231*
[Paraprevotellaceae] *Paraprevotella*	[Paraprevotellaceae] *[Prevotella]*
Cytophagaceae *Hymenobacter*	Cytophagaceae *Spirosoma*
[Weeksellaceae] *Chryseobacterium*	[Weeksellaceae] *Cloacibacterium*
Unclassified JG30-KF-CM45	Unclassified YS2
Unclassified Bacillaceae	Bacillaceae *Bacillus*
Bacillaceae *Geobacillus*	Unclassified Planococcaceae
Planococcaceae *Rummeliibacillus*	Planococcaceae *Sporosarcina*
Staphylococcaceae *Jeotgalicoccus*	Staphylococcaceae *Staphylococcus*
Unclassified Thermoactinomycetaceae	Unclassified Lactobacillales
Aerococcaceae *Abiotrophia*	Enterococcaceae *Enterococcus*
Unclassified Lactobacillaceae	Lactobacillaceae *Pediococcus*
Unclassified Leuconostocaceae	Clostridiaceae *02d06*
Clostridiaceae *Sarcina*	Unclassified EtOH8
Eubacteriaceae *Anaerofustis*	Eubacteriaceae *Pseudoramibacter_Eubacterium*
Lachnospiraceae *Epulopiscium*	Lachnospiraceae *Lachnobacterium*
Lachnospiraceae *Moryella*	Lachnospiraceae *Oribacterium*
Unclassified Peptococcaceae	Peptococcaceae *Peptococcus*
Peptococcaceae *rc4-4*	Peptostreptococcaceae *Clostridium*
Peptostreptococcaceae *Peptostreptococcus*	Ruminococcaceae *Clostridium*
Syntrophomonadaceae *Syntrophomonas*	Unclassified Veillonellaceae
Veillonellaceae *Acidaminococcus*	Veillonellaceae *Anaerovibrio*
Veillonellaceae *Mitsuokella*	Veillonellaceae *Selenomonas*
Veillonellaceae *Succiniclasticum*	[Mogibacteriaceae] *Mogibacterium*
Unclassified [Tissierellaceae]	[Tissierellaceae] *1-68*
[Tissierellaceae] *Anaerococcus*	[Tissierellaceae] *Finegoldia*
[Tissierellaceae] *GW-34*	[Tissierellaceae] *Gallicola*
[Tissierellaceae] *Peptoniphilus*	[Tissierellaceae] *Sporanaerobacter*
[Tissierellaceae] *WAL_1855D*	[Tissierellaceae] *ph2*
Unclassified SHA-98	Erysipelotrichaceae *Catenibacterium*
Erysipelotrichaceae *Coprobacillus*	Erysipelotrichaceae *Holdemania*
Erysipelotrichaceae *[Eubacterium]*	Erysipelotrichaceae *cc_115*
Fusobacteriaceae *Cetobacterium*	Fusobacteriaceae *Fusobacterium*
Leptotrichiaceae *Sneathia*	Unclassified Victivallaceae
Unclassified Caulobacteraceae	Caulobacteraceae *Brevundimonas*
Unclassified RF32	Unclassified Rhizobiales
Hyphomicrobiaceae *Hyphomicrobium*	Unclassified Methylobacteriaceae
Methylobacteriaceae *Methylobacterium*	Phyllobacteriaceae *Phyllobacterium*
Rhodobacteraceae *Paracoccus*	Rhodobacteraceae *Rubellimicrobium*
Unclassified Acetobacteraceae	Acetobacteraceae *Roseomonas*
Rhodospirillaceae *Novispirillum*	Sphingomonadaceae *Sphingobium*
Sphingomonadaceae *Sphingomonas*	Unclassified Alcaligenaceae
Burkholderiaceae *Burkholderia*	Burkholderiaceae *Lautropia*
Unclassified Comamonadaceae	Comamonadaceae *Comamonas*
Comamonadaceae *Curvibacter*	Comamonadaceae *Delftia*
Unclassified Oxalobacteraceae	Oxalobacteraceae *Cupriavidus*
Oxalobacteraceae *Oxalobacter*	Neisseriaceae *Neisseria*
Rhodocyclaceae *Dechloromonas*	Unclassified Desulfovibrionaceae
Unclassified 0319-6G20	Campylobacteraceae *Arcobacter*
Campylobacteraceae *Campylobacter*	Unclassified Aeromonadaceae
Enterobacteriaceae *Citrobacter*	Enterobacteriaceae *Erwinia*
Enterobacteriaceae *Morganella*	Enterobacteriaceae *Serratia*
Pasteurellaceae *Actinobacillus*	Pasteurellaceae *Aggregatibacter*
Moraxellaceae *Acinetobacter*	Moraxellaceae *Alkanindiges*
Moraxellaceae *Enhydrobacter*	Unclassified Pseudomonadaceae
Pseudomonadaceae *Pseudomonas*	Xanthomonadaceae *Stenotrophomonas*
Dethiosulfovibrionaceae *TG5*	Unclassified TM7-3
Unclassified F16	Unclassified Anaeroplasmataceae
Unclassified RF39	Unclassified ML615J-28
Verrucomicrobiaceae *Akkermansia*	Deinococcaceae *Deinococcus*

Table S3 Lesser Influential Taxa

Name (Family *Genera*)	
Unclassified Clostridiales	Unclassified Lachnospiraceae
Unclassified [Barnesiellaceae]	[Tissierellaceae] *Parvimonas*
Porphyromonadaceae *Parabacteroides*	Unclassified Erysipelotrichaceae
Ruminococcaceae *Faecalibacterium*	Alcaligenaceae *Sutterella*
Pasteurellaceae *Haemophilus*	Corynebacteriaceae *Corynebacterium*
Coriobacteriaceae *Eggerthella*	Veillonellaceae *Phascolarctobacterium*
Ruminococcaceae *Ruminococcus*	Coriobacteriaceae *Slackia*
Prevotellaceae *Prevotella*	Unclassified Gemellaceae
Dehalobacteriaceae *Dehalobacterium*	Ruminococcaceae *Anaerotruncus*
Lactobacillaceae *Lactobacillus*	Desulfovibrionaceae *Bilophila*
Desulfovibrionaceae *Desulfovibrio*	Bacteroidaceae *Bacteroides*
Turicibacteraceae *Turicibacter*	Unclassified Ruminococcaceae
Unclassified [Mogibacteriaceae]	Unclassified Christensenellaceae
Lachnospiraceae *Blautia*	Streptococcaceae *Lactococcus*
Oxalobacteraceae *Ralstonia*	Clostridiaceae *Clostridium*
Coriobacteriaceae *Adlercreutzia*	Streptococcaceae *Streptococcus*
Bifidobacteriaceae *Bifidobacterium*	Actinomycetaceae *Actinomyces*
Clostridiaceae *SMB53*	[Odoribacteraceae] *Butyricimonas*
Lachnospiraceae *Lachnospira*	

Figures

We plot in Fig. S1 marginal histograms of the square root relative abundance of taxa that most influence predictions but conditioned upon responder class. Histograms are paired by taxa, and each pair is plotted separately. The degree of distinction in distributions between subject groups varies across the taxa. Some trends are easier to recognize for certain taxa, such as *Roseburia* or *Ruminococcus*. However, differences in distribution shapes attributed to subject class are more difficult to identify for other taxa. Examples include *Anaerostipes* and *Veillonella*. Viewing the marginal distributions can complicate our ability to identify how the relationship between these taxa change according to responder status. With more data we could potentially develop a more effective way to identify how exactly the distributions of abundance differ.

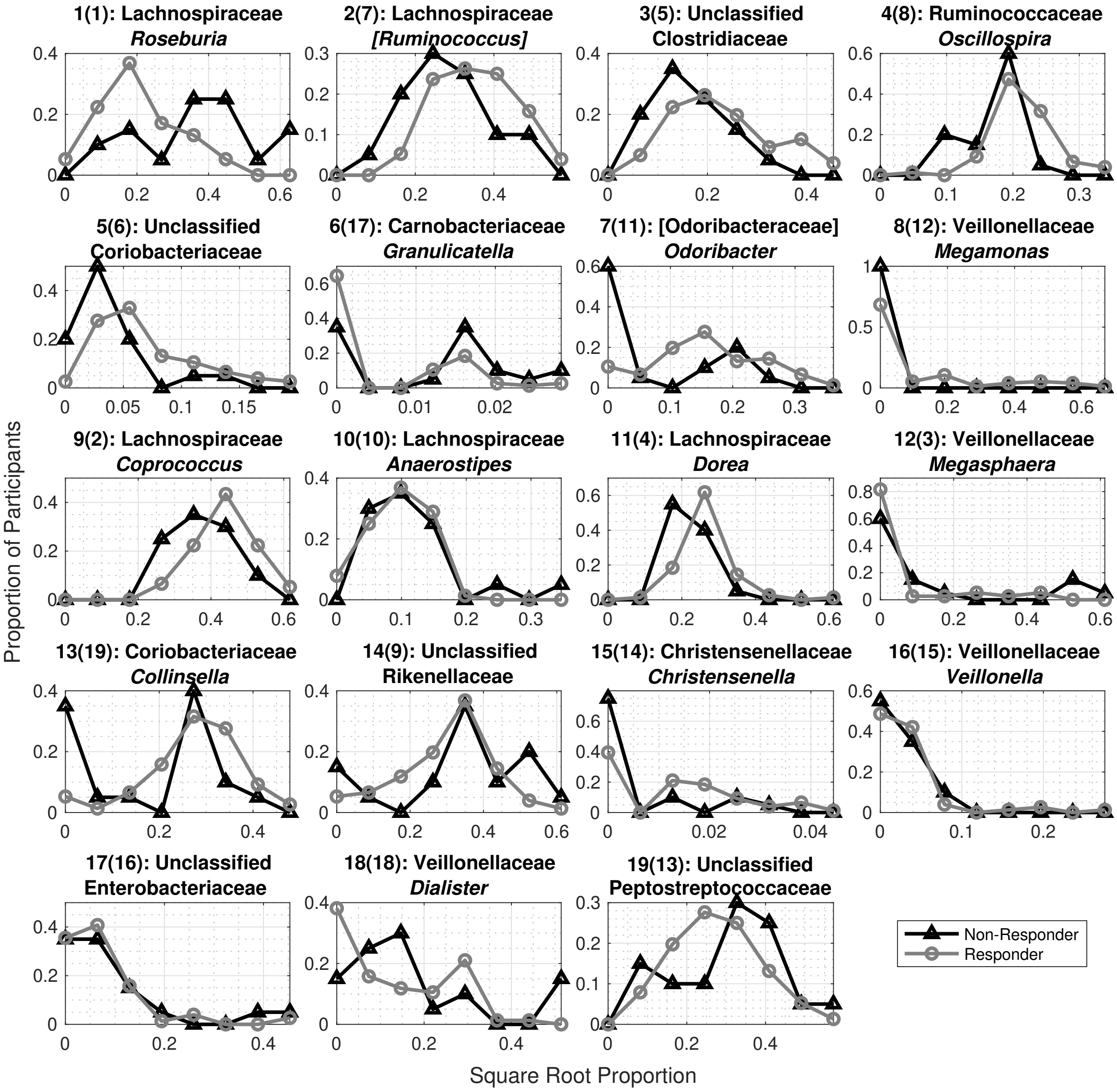

Fig. S1 Empirical distributions of square root sample proportions for the 19 most influential taxa conditioned upon responder class. The SVM determined a model that related relative abundances with responder status, so presumably there are identifiable differences in the joint distribution of relative abundances for either class. However, due to the high dimensionality, we present the marginal distributions, so important relationships between taxa are not displayed. Each histogram pair is labeled with its updated rank and its previous rank within parentheses. The taxa exhibited varying degrees of distinction between conditional distributions. Relative abundance of some taxa, such as *Anaerostipes* and *Megasphaera*, should be examined as a joint distribution with that of at least one other taxon to identify a distinguishing trend. Proportions of taxa, such as *Roseburia* and Reclassified *Ruminococcus*, were more directly correlated with responsiveness.
